# Association of Plasma Oligomerized Beta Amyloid with Neurocognitive Battery Using Korean Version of Consortium to Establish a Registry for Alzheimer’s Disease in Health Screening Population

**DOI:** 10.3390/diagnostics10040237

**Published:** 2020-04-20

**Authors:** Jung-Ju Lee, Youngki Choi, Soie Chung, Dae Hyun Yoon, Seung Ho Choi, Sung-Min Kang, David Seo, Kyung-Il Park

**Affiliations:** 1 Department of Neurology, Nowon Eulji Medical Center, Eulji University, Seoul 01830, Korea; sss331sss331@gmail.com; 2 Research and Development, PeopleBio Inc., Seongnam 13487, Korea; choi.youngki@peoplebio.net (Y.C.); kang.sungmin@peoplebio.net (S.-M.K.); seo.david@peoplebio.net (D.S.); 3 Department of Laboratory Medicine, Seoul National University Hospital Healthcare System Gangnam Center, Seoul 06236, Korea; soiec@snuh.org; 4 Department of Psychiatrics, Seoul National University Hospital Healthcare System Gangnam Center, Seoul 06236, Korea; dhyoon@snuh.org; 5 Healthcare Research Institute, Seoul National University Hospital Healthcare System Gangnam Center, Seoul 06236, Korea; cshmed@snuh.org; 6 Department of Neurology, Seoul National University Hospital Healthcare System Gangnam Center, Seoul 06236, Korea

**Keywords:** Alzheimer’s disease, blood biomarker, amyloid-β protein, oligomer, cognitive assessment, CERAD

## Abstract

The increasing prevalence of Alzheimer’s disease (AD) has become a global phenomenon presenting serious social and health challenges. For detecting early molecular changes in the disease, several techniques to measure varied species of amyloid beta in the peripheral blood have been recently developed, but the efforts to associate them with cognitive assessments have yet to produce sufficient data. We prospectively collected participants from the consecutive population who visited our center for brain health screening. In total, 97 participants (F:M = 58:39) aged 69.4 ± 7.52 were assessed. Participants performed the Korean version of the Consortium to Establish a Registry for Alzheimer’s disease (CERAD-K), the clinical dementia rating (CDR), plasma oligomeric amyloid-β (OAβ) level tests, routine blood tests, ApoE genotype, and brain MRI. Among total population, 55.7% had a CDR of 0, and 40.2% had a CDR of 0.5. The results showed that word memory and word recall, and the total scores of the CERAD-K were negatively correlated with the plasma OAβ level. With a cut-off value of 0.78 ng/mL for the OAβ level and a −1.5 standard deviation of age/sex/education adjusted norms for the CERAD-K; naming, word memory, word recall, word recognition, and total score were significantly correlated with the OAβ level. No correlation between the OAβ level and mini-mental status examination was found. Our results demonstrate that the level of plasma OAβ was well correlated with the measure of cognitive function through the CERAD-K in the field data collected from consecutive populations. Studies on longitudinal comparisons with large cohorts will further validate the diagnostic value of plasma OAβ as a useful biomarker for screening AD and predicting progression.

## 1. Introduction

Alzheimer’s disease (AD), one of the most common dementias, is a global health crisis. The number of people affected by the disease is projected to reach 13 million in the United States and 100 million globally by 2050 [1], with the prevalence rate increasing twofold every 20 years as a result of the aging population [2]. Symptoms of AD, mainly characterized by cognitive impairment, start 18 years before a clinical diagnosis can be made [3]. As a result of its renowned prolonged preclinical stage [4], consistent efforts are being made to identify and diagnose AD earlier. The most well-established and validated biomarkers for AD are the cerebrospinal fluid (CSF) biomarker test and positron emission tomography (PET) [5]. These biomarkers, however, have limitations in terms of being utilized as universal AD diagnostics as they are invasive, expensive, and often unavailable in many healthcare systems. Consequently, the development of a blood-based biomarker, which can provide minimally invasive, simple, inexpensive test procedures, and high patient accessibility, has become a sensational breakthrough for researchers and industries worldwide. Extracellular accumulation of amyloid-β (Aβ) and intracellular neurofibrillary tangles are the hallmarks of Alzheimer’s disease. When amyloid precursor protein (APP) is cleaved by both gamma and beta secretase, an amino acid peptide called beta amyloid (Aβ) is produced. Aβ may abnormally fold into oligomeric forms, fibrillary forms and finally into plaques. Research into Alzheimer’s disease has found that oligomerized Aβ (OAβ) are the most toxic among Aβ species [6,7] and are highly associated with the pathogenesis of AD [8,9]. Various efforts are being made to detect oligomerized Aβ in biofluid [10,11] with the aim of validating it as the long-anticipated blood-based biomarker for AD diagnosis. Multimer Detection System-Oligomeric Amyloid-β (MDS-OAβ) [12] is a technique which measures the level of the Aβ oligomerization (AβO) tendency after spiking purified synthetic Aβ into plasma samples. A previous study confirmed that the plasma OAβ level increases in plasma of AD patients but does not increase in the plasma of healthy normal control [12]. The increase in the plasma AβO level in AD highly correlates with CSF Aβ42 and Pittsburgh compound B (PiB) PET standard uptake ratio [13]. In addition, the elevated plasma OAβ level showed a strong correlation with cognitive performance in patients with AD, including an inverse correlation with scores on the mini-mental status examination (MMSE), cognitive abilities screening instrument, the common objects memory test, and a positive correlation with the Alzheimer’s disease assessment scale-cognitive portion scores [14]. The level of these biomarkers has been reported to correlate with cognitive performance, especially in patients within the mild cognitive impairment (MCI)-AD continuum, but this association is still controversial and it is unclear whether this correlation is found even in non-AD dementia. The Consortium to Establish a Registry for Alzheimer’s disease (CERAD), is a widely utilized neurocognitive assessment battery and is well validated in each country. Therefore, in this study, by comparing the MDS-OAβ value with the Korean version of Consortium to Establish a Registry for Alzheimer’s disease CERAD-K, we aim to validate that the plasma OAβ level reflects the cognitive and memory function of individuals using the Korean version of CERAD (CERAD-K), and the potential for utilization of MDS-OAβ as a feasible diagnostic method to screen people in the prodromal stage or MCI stage.

## 2. Materials and Methods

### 2.1. Study Population

We prospectively and consecutively collected 100 participants who had planned to undergo a brain examination as part of the health checkup and agreed to perform additional blood sampling for MDS-OAβ in our center. The institutional review board of the Seoul National University Hospital approved this study, and a written informed consent was obtained.

### 2.2. Plasma Oligomerized Beta Amyloid Assay

In all participants, blood samples (about 8 mL) were obtained in a heparin tube and immediately centrifuged at 1500× *g* for 10 min. Aliquots were stored at −70 °C until they were analyzed with an inBlood^TM^ oligomerized Aβ (OAβ) Test (Peoplebio Inc, Gyeonggi-do, Korea). This test utilizes a commercialized kit based on MDS-OAβ to quantify OAβ values. It is an atypical sandwich Enzyme-Linked Immunosorbent Assay (ELISA) using the epitope-overlapping antibodies specific for the N-terminus of beta amyloid (Aβ) to capture and detect plasma OAβ. The epitopes for the 6E10 and W0-2-HRP antibodies overlapped at the N-terminus of Aβ, and mouse monoclonal anti-6E10 (BioLegend, San Diego, CA, USA) and anti-W0-2-HRP antibodies (Absolute Antibody Ltd., Oxford, UK) were therefore used to capture and to detect OAβ, respectively.

Prior to the assay, aliquots of plasma samples were thawed at 37 °C for 15 min. All protocols were the same as in our previous papers [12,15,16]. As indicated in the assay protocol of the inBlood^TM^ OAß Test, PBR-1 (purified synthetic Aβ made by PeopleBio Inc.) was spiked into plasma and the mixture was incubated at 37 °C for 48 h. The incubated plasma sample mixture and serially diluted standard samples were added to each well of the plates. The plates were incubated at about 20 to 25 °C for 1 h. After washing three times with a washing buffer, the W02-HRP antibody was added to the wells, and the plates were incubated for 1 h at about 20 to 25 °C. To increase the sensitivity of detection, 100 μL/well of enhanced chemiluminescence substrate solution (Rockland Immunochemicals Inc., Limerick, PA, USA) was added, and the Relative Luminescence Unit (RLU) signal was detected using a Victor 3^TM^ multi-spectrophotometer. Dilutions providing signals in the linear range of the standard curves were used for the conversion to RLU values to determine the concentration of OAβ. Cut-off values for MDS-OAβ were set as 0.78 ng/mL [15].

### 2.3. Clinical Variables

The presence of hypertension was defined by the fact that the subject was taking hypertensive medication. The presence of diabetes mellitus was assumed if the patient was taking diabetes medication or showed HbA1c ≥ 6.5% at the time of the MRI visit. Hyperlipidemia was defined as LDL-cholesterol ≥ 160 mg/dl or total-cholesterol ≥ 240 mg/dl, or triglyceride ≥ 200 mg/dl at the time of visit. Information about smoking and alcohol drinking behavior was obtained based on routine questionnaires used at our center. Information about any history of hypertension, diabetes mellitus, and hyperlipidemia was also sought. At-risk drinking was defined according to The National Institute on Alcohol Abuse and Alcoholism (NIAAA) criteria [17]. Depression was screened through a Quick Inventory of Depressive Symptomatology-Self Report (QIDS-SR16), where equal or higher than 11 points indicated moderate to severe depression [18]. All subjects were interviewed and assessed clinically, including CDR and Global Deterioration Scale (GDS) tests by an experienced neurologist or psychiatrist. The CERAD-K test was performed on all subjects. The CERAD-K is composed of 8 subsets of cognitive function, which were verbal fluency, naming, mini-mental status examination (MMSE), word list memory, constructional praxis, word list recall, word list recognition, and praxis recall. The points from each subset were calculated according to its scoring system. Total II is a summed score excluding MMSE and Total I is a sum excluding both MMSE and the praxis recall score. For the dichotomized analysis, the abnormal CERAD-K in each subset and two types of total scores were defined as lower than −1.5 standard deviation of norms adjusting for age, sex, and education years. ApoE genotype testing was performed in 90 subjects. We also reviewed participants’ brain MRI and MRA including detailed hippocampal sequence.

### 2.4. Statistics

Student’s *t*-test was used for continuous variables, and the Mann-Whitney test was used unless the variables were normally distributed. Chi-square was used for di- or tri-chotomized categorical variables. Significance was set as *p* < 0.05.

## 3. Results

Among 100 participants, we excluded three persons who had MRI lesions potentially affecting the neuropsychological test outcome. Their lesions were traumatic cerebromalacia on the right frontotemporal area, anterior cerebral infarction on the left hemisphere, and sizable cerebellar infarction, respectively.

### 3.1. Aging Increases the Level of Oligomerized Beta Amyloid in Blood

**Figure 1:**
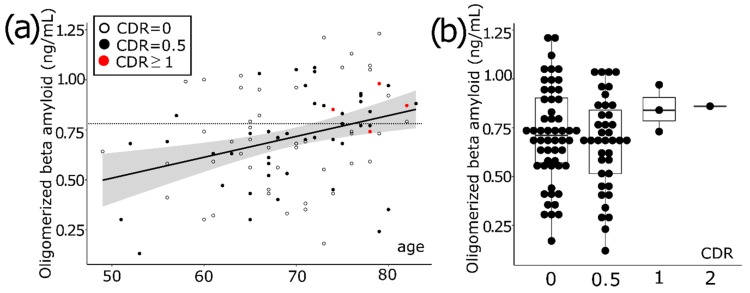
The distribution of plasma concentration of oligomerized beta amyloid (OAβ). (**a**) OAβ concentration increases with aging. The dotted line indicates a cut-off value of 0.78. CDR indicates the clinical dementia rating. (**b**) Most participants have a clinical dementia rating (CDR) of 0 or a CDR of 0.5. OAβ concentration was similar both in the CDR 0 and CDR 0.5 group.

Overall, 97 subjects (F:M = 58:39) were analyzed and their age was 69.4 ± 7.52. The plasma oligomerized Aβ (OAβ) level was 0.70 ± 0.24 ng/mL. The average education period was 13.56 years. Apoe4 carriers were 25 out of 90 persons (27.8%). The plasma OAβ level was strongly correlated with aging in our consecutive population (*r* = 0.33, *p* = 0.001) (Figure 1a). The number of subjects with clinical dementia rating (CDR) of 0 was 54 (55.7%), with a CDR of 0.5 was 39 (40.2%), with a CDR of 1 was two (2.1%) and a CDR of 2 was two (2.1%). The participants with a CDR of 0.5 met the criteria of MCI and the participants with a CDR of 0 were either cognitively healthy individuals or subjective cognitive decline (SCD) individuals. Taking into account that these participants were collected on the basis that they came to receive the brain examination, we found it rational to regard the whole group of participants with a CDR of 0 as SCD individuals. All subjects with a CDR of 0.5 showed 0.5 for the memory component score except one who obtained 1 for the memory score. Comparing groups between CDR0 and CDR0.5, the distribution of plasma OAβ level is not statistically different (Figure 1b).

### 3.2. Comparison of High vs. Low OAβ

Using a cutoff of 0.78 ng/mL [15], 63 subjects were categorized into the high plasma OAβ level group and 34 were categorized into the low plasma OAβ level group. Age, sex, education period, the presence of Apoe4 haplotype, hypertension, diabetes, smoking, at-risk drinking, and QIDS-SR16 were not statistically different between the two groups (Table 1). There are more people with high GDS scores in the high OAβ group (*p* = 0.03).

### 3.3. OAβ Is Well Correlated with the Consortium to Establish a Registry for Alzheimer’s Disease (CERAD-K) and its Subdomains

**Figure 2:**
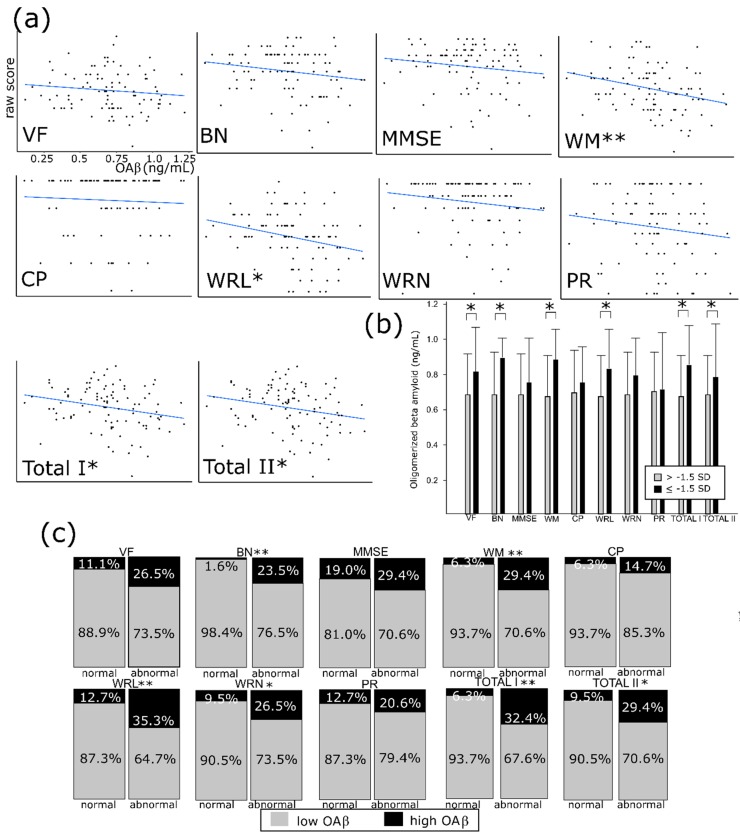
The subtests of the Korean version of Consortium to Establish a Registry for Alzheimer’s disease (CERAD-K) and plasma oligomerized beta amyloid (OAβ). (**a**) Raw scores of the CERAD-K and OAβ concentration. (**b**) The abnormal CERAD group (below −1.5 standard deviation of age/sex/education adjusting norms) in verbal fluency, naming, word memory/recall, and total scores showed higher OAβ concentration compared with control. (**c**) Abnormality in naming, word memory/recall/recognition, and total scores are significantly more frequent in high OAβ groups (≥ 0.78 ng/mL). SD—standard deviation; VF—verbal fluency; BN—Boston naming; MMSE—mini-mental status examination; WM—word list memory; CP—constructional praxis; WRL—word list recall; WRN—word list recognition; and PR—praxis recall. Total I is a sum of subtests except MMSE and PR and Total II is a sum except MMSE. * *p* < 0.05. ** *p* < 0.01.

The plasma OAβ level was negatively correlated with raw scores of words list memory (*r* = −0.29. *p* = 0.003), word list recall (*r* = −0.26, *p* = 0.008), total I (*r* = −0.24, *p* = 0.02), and total II (*r* = −0.23, *p* = 0.02) (Figure 2a). We compiled five groups based on age/sex/education adjusting norms: 1) 1st group: ≥ mean score; 2) 2nd group: mean~−1SD; 3) 3rd group: −1SD~−1.5SD; 4) 4th group: −1.5~−2SD; 5) 5th group: <−2.5SD. According to these categories, the domains which were significantly correlated with the plasma OAβ level were naming (*r* = 0.23, *p* = 0.02), word list memory (*r* = 0.32, *p* = 0.002), word list recognition (*r* = 0.24, *p* = 0.02), word list recognition (*r* = 0.23, *p* = 0.03), total I (*r* = 0.25, *p* = 0.01), and total II (*r* = 0.20, *p* = 0.047) (Figure 2b). The best correlated domain was the ‘word list memory’. This correlation was also evident when analyzing it in terms of dichotomized variables. We define “abnormal” as below −1.5 SD of sex/age/education adjusting norms in subtests as well as total score; the abnormality in four subtests, such as naming, word list memory/recall/recognition, and two types of total scores, was greater in the abnormal plasma OAβ group (*p* = 0.001, 0.005, 0.009, 0.03, 0.002, and 0.01) (Figure 2c). However, any methods of analysis regarding MMSE score did not demonstrate a significant correlation with plasma OAβ level (*p* = 0.14 for raw score, *p* = 0.36 for age/sex/education-adjusted interval and *p* = 0.25 for dichotomized analysis).

When we further analyzed the population with in the CDR 0 group, which is regarded as a cognitively healthy group, and the CDR 0.5 group, which is amnestic MCI separately, clinical variables such as age, the presence of hypertension, diabetes, hyperlipidemia, at-risk drinking, smoking, and education status were not statistically different between the groups (Table 2).

## 4. Discussion

The cerebrospinal fluid (CSF) biomarker test and PiB PET are excellent in terms of diagnostic accuracy, but there are limitations for these biomarkers in terms of being widely utilized in early diagnosis of AD due to the high invasiveness, high risk of side effects, and restricted availability to patients. Over the years, much effort has been devoted to global research and as a result several blood-based biomarkers have been developed [19,20]. These blood-based biomarkers have demonstrated high correlation between the level of Aβ42, Aβ40, and the risk of AD [21,22], but they require highly sensitive equipment to detect low-concentrated target proteins. On the other hand, MDS-OAβ utilizes spiking of purified synthetic Aβ into plasma and incubation thereafter to measure the level of AβO tendencies in plasma [12]. The comparative measurement on the level of AβO tendencies in AD and normal plasma using MDS-OAβ was conducted and the resulting values were 1.43 ± 0.30 ng ml and 0.45 ± 0.19 ng/mL, respectively, showing the significant difference between the two groups (*p* < 0.001) [15]. Reference interval was induced and the cut-off value was validated according to the guideline of Clinical Laboratory Standard Institute C28-A3, the 95% reference interval applied to the normal group was 0.783 ng/mL and it was very close to the cut-off value of receiver operating characteristic (ROC) curve analysis. Applying the cut-off of 0.78 ng/mL, area under curve (AUC) was 0.999, sensitivity was 100% and specificity was 92.3%, showing high accuracy in terms of AD diagnosis [15]. In this study, the MDS-OAβ value positively correlated with age, contrary to the results showing no correlation with age in community-based normal controls [15]. This difference might be due to the fact that most people who came to the healthcare center and participated in this study voluntarily were potentially SCD population. This also seems to affect the result that the level of OAβ is not significantly different both in the groups of CDR = 0 and CDR = 0.5.

The quality of this study lies in the fact that it validated the relationship between MDS-OAβ and the CERAD-K, a Korean version of the standardized neuropsychological evaluation and diagnosis of AD [23]. Rossetti et al. [24] described the CERAD total score in AD patients, and the normal control for four years showed a significantly greater decrease in AD patients than in the normal group. Hwang et al. [25] confirmed that the CERAD scores decreased as the disease progressed through three years of follow-up of participants in the cognitive normal, SCD, MCI, and ADD groups, and CSF Aβ42 levels in each group also decreased in correlation. In 2019, Meng et al. [14] described that an increase in MDS-OAβ levels in AD patients correlated with episodic memory loss. Other studies have reported that scores on language-related evaluative tasks such as word list memory and word list recall reflect AD-associated cognitive and memory function changes [25,26]. Furthermore, the decrease in the CERAD score in the order of normal individuals, MCI, and AD correlated with the atrophy of white matter lesions and gray matter in the brain [27], and scores for the word list recall and memory from the CERAD had a close relationship with atrophy in the medial temporal lobe [28]. In addition, the study by Youn et al. [16] confirmed that the correlation between the change in the MDS-OAβ value and brain atrophy in the form of AD exists. The cross-sectional and longitudinal evidence of connections among neurodegeneration, blood OAβ, and cognitive impairment in predementia status should be sought in future studies.

The significance of this study lies in the fact that the results are based on field data collected from the consecutive population. However, the main limitation is the small number of participants in the study. We understand the necessity of future studies using more refined global cognitive measures, larger samples with non-AD dementias, and ultimately a longitudinal design to establish the mechanisms for cognitive impairment caused by AD. The longitudinal studies of test-based cognitive performance assessments, along with blood biomarkers for people at high risk of dementia, are crucial to understanding cognitive decline mechanisms and improving AD diagnostic accuracy in the future.

## Figures and Tables

**Table 1:** Demographics and clinical data of the population of high and low group of plasma OAβ levels.

	High OAβ	Low OAβ	*p* Value
Age	72.62 ± 7.00	67.67 ± 7.27	0.02
Sex (Female %)	20/34 (58.8)	38/63 (60.3)	0.89
Education (yrs)	14.06 ± 3.26	13.30 ±4.05	0.34
CDR			0.24
CDR = 0	19/34	35/63	
CDR = 0.5	12/34	27/63	
CDR ≥ 1	3/34	1/63	
GDS			0.03
GDS = 1	18/34	34/63	
GDS = 2	10/34	28/63	
GDS ≥ 3	6/34	1/63	
Apoe4 carrier (%)	10/32	15/58	0.59
Hypertension	12/34	29/63	0.26
Diabetes	6/34	12/63	0.39
Hyperlipidemia	21/34	40/63	0.39
Smoking			0.25
Never	25/34	46/63	
Ex	8/34	13/63	
Current	0/34	4/63	
At-risk drinking	2/25	13/58	0.21
QIDS-SR(cutoff ≥ 11)	2/31	4/57	0.09

OAβ—oligomerized amyloid beta; CDR—clinical dementia rating; GDS—global deteriorating score; QIDS-SR—Quick Inventory of Depressive Symptomatology-Self Report.

**Table 2:** Clinical variables of population with CDR = 0 and CDR = 0.5.

	CDR = 0 (*n* = 54)		CDR = 0.5 (*n* = 39)	
Age	69.07 ± 7.14		68.95 ± 7.90	
	High OAβ (*n*)	*p* value	High OAβ (*n*)	*p* value
Hypertension	6/25	0.11	5/14	0.72
Diabetes	4/12	1.00	2/6	1.00
Hyperlipidemia	11/35	0.43	9/25	0.48
Problem drinking	2/9	0.65	0/6	0.30
Smoking		0.74		0.46
None	14/40		10/29	
Ex	5/13		2/7	
Current	0/1		0/3	
QIDS-SR (≥moderate)	2/7	0.62	2/9	0.69
Apoe4 carrier	6/15	0.70	2/8	1.0
Education (<12 yrs)	4/10	0.73	2/8	1.0
